# Assessment of the effect of application of an educational wiki in flipped classroom on students’ achievement and satisfaction

**DOI:** 10.1186/s12909-020-02223-0

**Published:** 2020-09-07

**Authors:** Ali Ahmadian Khoynaroud, Amirhossein Akbarzadeh, Morteza Ghojazadeh, Saeideh Ghaffarifar

**Affiliations:** 1 Education development center, E-learning center, Tabriz university of Medical Sciences, Tabriz, Iran; 2 Medical Education department, Tabriz University of Medical Sciences, Tabriz, Iran; 3 Research Center for Evidence-Based Medicine, Health Management and Safety Promotion Research Institute, Tabriz University of Medical Sciences, Tabriz, Iran; 4 Medical Education Research Center, Health Management and Safety Promotion Research Institute, Tabriz University of Medical Sciences, Tabriz, Iran

**Keywords:** Collaborative learning, Distance education and online learning, Twenty-first century abilities, Architectures for educational technology system

## Abstract

**Background:**

Active participation in group assignments is an invaluable way to realize collaborative learning; however, there are several challenges attributed to the traditional way of doing group assignments. This study explores the synergistic effects of flipped classrooms and a wiki-authoring group activity on students’ learning outcomes and the quality and quantity of their group-work.

**Methods:**

In this action research, 205 master students of a medical school were involved in a course blended with flipped classrooms. While learning from online and in-class activities, students did their group assignment on an educational wiki (*n* = 85) or in a conventional way (*n* = 120). Assessment in this study was done in both formative and summative ways. Formative assessment included quizzes at the beginning of each class and students’ self-assessment (focused on their satisfaction with different educational activities of the course, using an 11-item validated satisfaction questionnaire). The summative assessment incorporated assessment of the quantity and quality of students’ participation in doing group assignments(by a five-item checklist); quizzes at the end of each class; the final exam; assessment of students’ competency in transferring their learning into creating an outline for a hypothetical article and writing topic sentences. Using SPSS 21.0 and employing independent samples t or Mann –Whitney U tests, the educational impacts of the course were compared in two groups.

**Results:**

Students in the wiki-group were more satisfied with the course. Both quantity and quality of the group assignment among students in the wiki group outweighed those among the students in the non-wiki group. Univariate linear regression analysis of the models between students’ satisfaction with flipped classrooms and the quality of their participation in doing their group as well as their attitude towards the group assignment showed that the changes in the quality of the wiki students’ group assignment and their attitude were dependent on the changes of their satisfaction with flipped classrooms.

**Conclusions:**

The findings of this study confirm that a wiki-authoring group assignment is effective in achieving student learning outcomes and integrating a wiki with flipped classrooms increases wiki accomplishment. Collaborating on a wiki activity improves both quality and quantity of group assignments among students.

## Highlights

- Collaborating on a wiki reduces the challenges of doing group assignments.
- A wiki-authoring group assignment enhances students’ attitudes towards their group assignment.
- A wiki-authoring group assignment is effective in achieving student learning outcomes.
- Collaborating on a wiki activity improves quality of group assignments among students.
- Integrating a wiki with flipped classrooms increases wiki accomplishment.

## Background

Students’ collaborative and individual learning are positively related to each other [1]. Active participation in group assignment (GA)s is an invaluable way to realize collaborative learning [2]; however, there are several challenges attributed to the traditional way of doing GAs [3]. Traditionally, teachers have a poor supervision over students’ activities during the course and no feedback is provided on their work by the course teacher. Furthermore, just one or two students usually complete the GA on behalf of all group members and teachers usually assess students’ works just at the end of the course. Because of the poor supervision and late assessment, the exact quantity and quality of participation of each student in the GA is not specified and the same scores are given to all students in the group.

In order to decrease the challenges attributed to the traditional way of doing GAs and to respect millennials’ appreciation for technology [4],the opportunities for collaborative learning are provided through educational wikis are in some of the leading universities in the United Kingdom, America, Canada, Switzerland, New Zealand and Germany [5]. Wiki is a platform composed of one or more web pages connected to each other. People can write on and store information freely on it. They can also edit their own or others’ content on a wiki. Engaging in a wiki activity, students transfer their learning into a common work. While learning independently in accordance with their own learning style, they learn from the peers as well [6]. Working on a wiki can facilitate group collaboration, knowledge acquisition and writing skills of participating students [7].

Although, wikis are extensively adopted in education recently and their worth in collaborative learning is confirmed, it was concluded in an integrative review that wikis’ effectiveness in “achieving student learning outcomes” is uncertain and further effectiveness assessment studies are needed. Moreover, based on the findings of another study at the Victoria University of Wellington, wiki alone is not enough to enhance the attitude of the students towards their group assignment [8]. Because of the informal nature of a wiki activity, students may share imprecise and less-academic information on it. In order to increase the effectiveness and quality of the group activity on a wiki, it is recommended that facilitation of critical judgment be considered in the design of the GAs and students be familiar with the way of accessing the necessary sources [8]. Integrative application of wikis; electronic contents (podcasts); web log activities such as discussions on a forum and student-tutor interactions are recommended in a research. According to the research, synergistic contributions of the above- mentioned elements may enhance students’ learning and may provide “a coherent wholesome learning experience” for them [9].

Considering the suggestions to investigate the learning values of wikis combined with other educational interactions, some questions raise in mind: Is a wiki-authoring group assignment effective in achieving student learning outcomes? Does a wiki-authoring GA enhance students’ attitude towards their GA? Does integrating a wiki with flipped classrooms increase wiki accomplishment?

To answer these questions, this action research intends to explore the synergistic effects of a wiki-authoring activity integrated with flipped classrooms on 205 master students’ learning outcomes and the quality and quantity of their group-work. It is hypothesized that using wiki on a FC will promote students’ attitudes towards their wiki-supported GA and increase both quality and quantity of their GA as well.

## Methods

### Research design and setting

This study was an action research, in which, researchers and participants collaboratively work through an iterative process to define a problem, to design an action based on the participants’ needs, to carry it out and discover the changes [10]. An action research is “a cyclical process of reflective practice particularly suited to educational settings” [11]. It was conducted to explore the impacts of a course blended with flipped classrooms on both the attitude of master students towards their wiki-supported GA and the quality of their GA.

It took place at the education development center affiliated with our university of medical sciences in six consecutive semesters, from September 2016 to December 2019. All the students of 4 years were included in the study. Conducting the study was approved by the university ethics review board (N: IR.TBZMED.REC.1396.1129).

### Participants

Participants were master students at the School of Medicine. All students had to pass a course entitled: “Writing and presenting articles in English”, in the first two semesters of their study at medical school. During four semesters, 205 master students enrolled in this course. As passing the course was compulsory for all master students, all enrolled students were included in the study. Participating students were studying in eight different disciplines: physiology, biochemistry, bacteriology, virology, anatomy, immunology, hematology and medical education. All students, who had not previously passed a similar course, were included in the study. Later, the data about the students who had not completed the satisfaction questionnaire were set aside and only the data on 171 remaining students were analyzed.

### Intervention

All students were involved in a blended course, with both online and in-class parts. While learning from online and in-class activities, students were asked to do a GA. They were free to choose one of the two available options for doing their GA. Those options were doing the GA through an educational wiki or doing it in a traditional way. Regardless of their selected option, all students were informed that 40% of their overall score would be allocated to their GA. Students in both groups had to follow all in-class and out-of- class activities. The only difference between the two groups was in the form of their GA; therefore, the results of the study will be classified into two groups of wiki or non-wiki.

### Out of class activities of the course

This part was delivered through an open-source and freely available Moodle (Modular Object-Oriented Dynamic Learning Environment) [12]. The version of employed Moodle was 3.0.4 [13].. An orientation session was held to ensure that all students can manage all predetermined activities on the platform.

Using the iSpring Suite 8 software, twelve electronic- contents (e-content), in the form of the sound synchronized with PowerPoint slides, were produced and uploaded into the platform. Those contents were standard in format and the students could control them on their own players (on a personal computer or a smartphone). The e-contents covered all the learning objectives of the course and students could use them many times, in their desired time and place.

The expected online activities included studying e-contents of the course and discussion on a forum and the students in the wiki group had to do their GA on line, as well.

### In-class activities of the course

In this part, students attended flipped classrooms (FCs). The same teacher conducted flipped classes in both wiki and non-wiki groups. She was an MD-PhD, who was trained at a master program in medical education. She was the instructor of flipped classrooms at the university and national level. The teacher’s performance was assessed by department head, learners of the course and peers at scholarship committee as well. Furthermore, this educational intervention has been criticized by peers at university, leading to a learning and teaching scholarship award in 2018(Number: 5/d/214008). Students were divided into groups of four. They were asked to summarize the content of each session in their group and bring a one-page summary to the class. They were also requested to be prepared for a 15-min in-class presentation. They were explained that each session they should take on different roles (presenter, note taker or writer) in the group.

Details for students activities in each FC, divided by in or out of class activities, according to their implementation order are summarized in Table 1**.** In the flipped classrooms, the teacher did not provide direct instruction. S/he only facilitated students’ learning through discussion on the introduced e-contents of the classes; providing a supportive environment for students’ collaborative learning and cognitive development; incorporating formative and summative assessment into each session of the class; providing mini-lectures to clarify any misunderstandings; and mapping out group assignments [14, 15]. Playing these roles are justified by constructivist and cooperative learning theories [11].

**Table 1:** Details for students activities during a semester, divided by in or out of class activities, according to their implementation order

	Order	Type of activity	Description	Duration
**Online (out-of class) activities**	1	Self-regulated learning	Downloading and listening to the e- contents, whenever and wherever students liked (flexible asynchronous e-learning)	Variable according to students’ interest and need
	2	Group discussion in a forum	Raising questions in the forum and discussion with each other (peer-assisted learning)	
**in-class activities (at flipped classrooms)**	3	Quiz at the beginning of the class	Discovering any possible misunderstandings of the content (formative assessment)	10 min
	4	Summary sharing by students	Sharing a brief one-page summary of the e-content of that session with other groups (collaborative learning)	10 min
	5	Student presentations	A 15-min presentation by one group (Peer education)	15-min
	6	QA & Group discussion	Questions and answers from students and facilitation by the teacher	35 min
	7	Micro lecture by the teacher	Summarizing and closing the session	10 min
	8	Quiz at the end of the class	Summative evaluation	10 min

### Wiki activity

The version of the wiki, used in this research, was 2,015,111,600 [13] and its programming language was PHP (Hypertext Preprocessor) [16]. It had already been installed with the Moodle package and had a simple markup formatting. Students in the non-wiki group, who did their GA in the traditional way, did not have access to the wiki on the LMS (Learning Management System).

The wiki had various features including, writing and editing text, importing and editing images, importing audio and video files, drawing tools, drawing and editing tables, hyperlinking, inserting and editing statistical formulas, structuring and organization of the text.

“Both the course teacher and the IT expert, who was working at virtual training center of the university, trained the students for wiki”. Through a pilot study, wiki’s features were improved based on the feedback from eight peer students and five peer researchers. Reflections by the research team were helpful as well. After the modifications, the features of search, spell check, insertion of emotions and email notification were added to the wiki too.

The learners’ GA was focused on a collaborative writing about do’s and don’ts in writing the articles in English. Applying the above mentioned features of the wiki, students were able to start writing, editing and structuring from everywhere in the text. They could also structure their group writing with the subject division hierarchical method. Each semester, the wiki was available for only the students of that term; therefore, students could contribute to the web pages of their own group.

The course teacher (SGH) was able to supervise LMS activities of all learners 24 h a day. By controlling history and logs of the learners, she was able to assess both the quantity and the quality of each student’s activity on the LMS and wiki; therefore, it was possible to differentiate the various activities of the students in the system. Different activities of students included a simple log in, moving between pages or typing and editing in the wiki. Any changes made by the students in their writing would be highlighted in the text and the highlights were evaluated by the teacher. Both the quantity and quality of each student’s participation in doing the group assignment were assessed by a five-item checklist (Additional file 2), which is described later under the heading of “Evaluation of the intervention and data analysis”.

When the quantity or quality of a student’s activity was not desirable, the teacher sent an email to her/him via the LMS. Since, according to the university regulations, the minimum passing score in this course was 14 out of 20, the minimum acceptable score for both the quantity and quality of students’ participation was considered to be 3.5 out of 5. A reminder notice email was sent to students with a participation score of less than 3.5.Drawing figures of “content access”, “number of active participants” and “hits distribution”, the teacher realized passive or very active students. Those figures also helped the teacher figure out which contents were not accessed by every student. All students were informed that 40% of their overall score would be allocated to their GA.

### Evaluation of the intervention and data analysis

Assessment in this study was done in both formative and summative ways. Formative assessment was performed in the form of a quiz at the beginning of the class. In this type of assessment, students’ knowledge was evaluated in order to identify and eliminate any misunderstandings about the content of the course, which had already been provided electronically. To do this, students answered 10 multiple choice questions (MCQs) on the LMS and received the necessary feedback immediately. The correct answers to the questions were shown to them at the end of the test, and in cases where the mistakes were repeated by a large number of students, the teacher gave more explanations about those common misunderstandings at the end of each class.

The quiz at the end-of-the class was similar to the one at the beginning. It included ten MCQs too. The only difference between these two quizzes was the summative nature of the latter one. Unlike the first quiz, the score of the second one was considered to be part of the students’ final score. The students were previously informed about this point.

The summative assessment in this study included several parts: First, students self-assessed their satisfaction with different educational activities of the course. They also specified the amount of their learning from each component of the course. An 11-item satisfaction questionnaire was designed and validated for the present study. To assess the content and face validity of the satisfaction questionnaire, ten experts in the field of medical education evaluated the necessity, relevance, appropriateness and clarity of the items [17, 18] and 6 master students examined any ambiguity in understanding of the items [17, 18]. Internal consistency of the items was assessed by calculating Cronbach’s Alpha and Intra-class Correlation Coefficient (ICC) was calculated to judge the stability of the results [18]. For doing so, 30 master students completed the questionnaires twice with a two-week interval.

Students’ satisfaction with each item of the questionnaire was specified, employing a five-point Likert scale (Very high, above average, Average, below average, Very low). Students specified their attitude toward performing their group, responding to 5 questions with the same Likert scale. All the items of students’ satisfaction questionnaire are presented in Additional file 1.

Students’ learning was assessed in two ways: First, as a formative assessment, both the quantity and the quality of each student’s participation in doing their GA were continually evaluated during the semester. At the end of the semester, the quantity and quality of each student’s participation was rated in a range from zero to five, using a five-item checklist. The checklist to assess the quantity and quality of each student’s participation is attached as Additional file 2. The criteria to assess the quality included completeness, accuracy and pertinence of the content, which is written by each student. The usage of pertinent terminologies and the quality of the links established between the pages developed by other students and their own pages were used to assess the quality of each student’s participation too.

Second, students’ average score in their summative exam was used to assess their learning. The summative exam included extended matching items, multiple choice questions and fill-in- the blanks items. The minimum passing score for this summative exam was 14 out of 20 and students should have achieved at least 14 to be successful in it.

Students’ competency in transferring their learning into creating an outline for a hypothetical article and writing topic sentences for each part of that article was used to assess the impact of the course. That competency was examined in students’ summative exam.

In all, calculation of students’ overall score for the course was done as: 40 % of the score was allocated to the quantity and quality of student participation in doing group assignments; 20 % to the quizzes at the end of each class; 30 % to the final exam and 10 % to their competency in transferring their learning into creating an outline for a hypothetical article and writing topic sentences.

In order to regard some unanticipated consequences of the wiki-authoring activity, all learners were asked to critique the process of their co-construction activity as well. Students’ feedback together with their aliases was emailed to the course teacher by the class representative immediately at the end of the semester.

### Statistical analysis

Students’ satisfaction with each component of the education; their overall self-declared satisfaction with the whole course; their average score in the summative exam of the course; quantity and the quality of each student’s participation in doing their GA and also their attitudes toward performing their group were described in wiki and non-wiki groups, reporting proper descriptive statistics.

Employing independent samples t test or Mann –Whitney U test, the results of the course were compared in two groups. As the distribution of some variables was not normal, they were first transformed to their natural logs. Then, the correlation between the mean score of students’ satisfaction with flipped class rooms and the mean score of the quality of each student’s participation in doing their GA as well as their mean score of their attitudes towards their GA were investigated, using Spearman’s rank correlation coefficient. The causal associations between those variables were evaluated by univariate linear regression analysis. The data were analyzed Using SPSS for windows version 21.0. Significant meaningful differences were reported based on the Asymp.Sig. (2-tailed). Values of P less than 0.05 were considered statistically significant.

## Results

During six academic semesters, 205 master students of medical sciences took part in this action research and 85 of them voluntarily chose to have wiki activities. In all, 70 out of 85 students in the wiki group and 101 out of 120 in the non-wiki group completed the satisfaction questionnaires (response rates: 82.4,84.2%, respectively). Later, only the data about 171 respondents were analyzed.

The mean age of students in the wiki and non-wiki groups were 24 ± 2 and 25 ± 3 years, respectively. Women accounted for 75% in the wiki and 65% in the non-wiki groups.

The satisfaction questionnaire was validated by the active participation of 10 experts and 6 master students. Content Validity Ratio (CVR) and Content Validity Index (CVI) of the questionnaire were 0.71 and 0.87, respectively. The satisfaction questionnaire demonstrated an Impact Score = 3.81, Cronbach’s α = 0.83 and ICC = 0.724 (*p* < 0.00).

Students’ satisfaction with each component of the education in both groups is presented in Table 2**.** Based on the self-assessment results, the mean score of the students’ overall satisfaction in the wiki group was 40.30 ± 3.70; while it was 37.23 ± 3.00 in the non-wiki group (*P*-value = 0.000).

**Table 2:** Description^a^ of students’^b^ satisfaction with each component of the education in a course blended with flipped classrooms, divided by their collaboration way in doing their group assignment

Component of education	Satisfaction item	Wiki-group(***n*** = 70)	Non-wiki group(***n*** = 101)
**E-contents**	Fitness of the content of e-contents to my needs	3.60 ± 0.88	3.70 ± 0.93
	The quality of e-contents	3.76 ± 0.82	3.71 ± 0.92
**Forum**	The usefulness of forum discussions	2.60 ± 0.65	2.67 ± 0.63
	User-friendliness of the forum discussions	2.76 ± 0.75	2.56 ± 0.71
**Flipped classroom**	The effectiveness of the in- class discussions	3.36 ± 0.68	3.33 ± 0.69
	Possibility to participate in class discussions	4.17 ± 0.64	3.96 ± 0.58
	The worth of summary sharing in learning	4.07 ± 0.62	4.04 ± 0.49
	The value of student presentations in learning	4.03 ± 0.54	4.02 ± 0.55
	The role of teachers’ brief lecture	4.20 ± 0.73	4.00 ± 0.85
**Group assignment**	Appropriateness of the group assignment	3.96 ± 0.84	2.65 ± 0.71
	The helpfulness of the group assignment	3.80 ± 0.81	2.57 ± 0.78
**Attitude towards the group assignment**	The value of group assignment in motivating me to learn more	3.81 ± 0.84	2.62 ± 0.72
	The impact of the group assignment to increase my confidence in writing an article	3.69 ± 0.97	2.56 ± 0.62
	The worth of the group assignment in identifying my weaknesses and strengths in writing articles	3.66 ± 0.92	2.53 ± 0.67
	The importance of the group assignment experience in building my teamwork skills	3.64 ± 0.82	2.69 ± 0.64
	The role of the group assignment in encouraging me to participate in similar experiences	3.81 ± 0.84	2.78 ± 0.72
**The whole course**∞	**Satisfaction with the whole course (self-declared)**	3.84 ± 0.81	3.38 ± 0.79

^a^All numbers describe Mean ± SD of satisfaction with each item

^b^ Master students in medical school, who did their group assignment during a semester based on a wiki or face-to- fact collaboration

The effects of the course on the learning and satisfaction of the students in both groups are compared in Table 3.

**Table 3:** Comparison of the effects of a course, blended with flipped classrooms, on students’^a^ scores in wiki (n = 70) with non-wiki (n = 101) groups

Result measure	Group	Mean ± SD^c^	Normal Range of mean	Mean Rank	Significance
**Number of e-contents, downloaded and studied by students**	Wiki	10.90 ± 1.00	0 to12	96.23	0.019
	Non-wiki	10.51 ± 1.08		78.91	
**Score of the quiz at the beginning of the class**	Wiki	3.36 ± 0.78	0 to 5	90.79	0.243
	Non-wiki	3.22 ± 0.72		82.68	
**Score of the quiz at the end of the class**	Wiki	4.09 ± 0.65	0 to 5	92.24	0.118
	Non-wiki	3.93 ± 0.62		81.67	
**Score of the quantity of the participation in doing the group assignment**	Wiki	3.54 ± 0.50	0 to 5	^b^119.60	< 0. 001^e^
	Non-wiki	2.67 ± 0.57		^b^ 62.71	
**Score of the quality of the participation in doing the group assignment**	Wiki	4.11 ± 0.65	0 to 5	^b^ 125.61	< 0. 001 ^e^
	Non-wiki	2.86 ± 0.66		^b^ 58.54	
**Final exam score**	Wiki	16.59 ± 1.04	0 to 20	^b^ 124.76	< 0. 001 ^e^
	Non-wiki	14.52 ± 1.38		^b^ 59.13	
**Score of competency in writing an outline and topic sentences for a hypothetical title**	Wiki	16.51 ± 1.32	0 to 20	^b^ 132.11	< 0. 001 ^e^
	Non-wiki	12.25 ± 2.35		^b^ 54.04	
**Score of satisfaction with uploaded e-contents**	Wiki	7.36 ± 1.38	2 to 10	84.10	0.665
	Non-wiki	7.42 ± 1.50		87.32	
**Score of satisfaction with forum activities**	Wiki	5.36 ± 1.10	2 to 10	90.05	0.353
	Non-wiki	5.24 ± 1.04		83.19	
**Score of satisfaction with flipped classrooms**	Wiki	19.83 ± 1.58	5 to 25	95.76	0.028^d^
	Non-wiki	19.35 ± 1.49		79.23	
**Score of satisfaction with group assignment**	Wiki	7.76 ± 1.41	2 to 10	^b^ 126.19	< 0. 001 ^e^
	Non-wiki	5.23 ± 1.25		^b^ 58.14	
**Score of satisfaction with the whole course**	Wiki	40.30 ± 3.70	11 to 55	^b^ 114.88	< 0. 001 ^e^
	Non-wiki	37.23 ± 3.00		^b^ 65.99	
**Score of the attitudes towards the worth of the group assignment**	Wiki	18.61 ± 3.46	5 to 25	^b^ 125.34	< 0. 001 ^e^
	Non-wiki	13.20 ± 2.26		^b^ 58.73	

^a^ Master students in medical school, who did their group assignment during a semester based on a wiki or face-to- fact collaboration

^b^ Variables, whose distribution was not normal

^c^About variables, whose distribution was not normal, Mean ± SDs are written just for the ease of the comparison

^d^ Significance of independent samples t test

^e^ Significance of Mann-Whitney U test

Based on the results presented in Tables 2 and 3, students in the wiki group were more satisfied with the course and flipped classrooms. Collaborating on the wiki, they found their GA more appropriate and helpful compared to their counterparts in the non-wiki group. According to the participants, compared to the traditional way of doing GA, doing group assignments on the wiki more motivated students to learn. Doing GA on the wiki had better impacts on increasing students’ confidence in writing an article. By group working on a wiki, students identified their weaknesses and strengths in writing articles more and they were more encouraged to participate in similar experiences in the future. In addition, working on the wiki helped students to build better teamwork skills. The number of e-contents downloaded and studied by students in the wiki group was more than the number in the non-wiki group. The mean of scores of the quiz at the beginning of the class; the quiz at the end of the class; quantity and quality of their participation in doing the GA; final exam and competency in writing an outline and topic sentences for a hypothetical title among students in the wiki group outweighed those among the students in the non-wiki group.

In the wiki group, students performed their GA on a common wiki. The range for their completed work in each semester was 34 to 48 pages. Eighty seven percent of the students received feedback, on both the quantity and quality of their participation. The mean time of the constructive feedback provided by the teacher was three hours for each student. Students in non-wiki group submitted their GA to the course teacher at the end of the semester. Although all students were requested to ask for the teacher’s feedback every time during the term, those in the non-wiki group never asked for her/his feedback. The range for the completed work in this group was 15 to 23 pages. The number of the pages of the students’ group work was considered as an objective indicator for the quantity of the effort they put on their team work. It also provided an answer to the question of which group of students was most motivated to collaborate on their group work and to learn more.

Neither students in the wiki, nor those in the non-wiki welcomed discussions in the forum. Eleven and eight questions and answers (Q&As) were presented in the forum in the wiki and non-wiki groups, respectively.

Students’ satisfaction with flipped classrooms correlated at r = 0.485(Sig = 0.017) with quality of their participation in doing their GA for 70 students in the wiki group. That Spearman’s rho was − 0.200(Sig = 0.045) for 101 students in the non-wiki group.

The correlation of students’ satisfaction with flipped classrooms with their attitude towards their GA was r = 0.508(Sig = 0.000) and − 0.234(Sig = 0.019) in the wiki and non-wiki groups, respectively.

Table 4 represents the summary and parameter estimates of the models, in which students’ quality of participation in doing their GA and their attitude towards their GA are dependent on their satisfaction with flipped classrooms.

**Table 4:** Model Summary and Parameter Estimates of the Model between students’^c^ satisfaction with flipped classrooms; quality of their participation in doing their group assignment and their attitude towards the group assignment

Model		Unstandardized Coefficients		Standardized Coefficients	t	*P-Value*
		B	Std. Error	Beta		
1 _a_	(Constant)	**0.35**	**0.29**		**1.17**	**0.24**
	Satisfaction with flipped classrooms	**0.63**	**0.23**	**0.20**	**2.77**	**0.006**
2 _b_	(Constant)	**−0.20**	**0.34**		**−0.58**	**0.56**
	Satisfaction with flipped classrooms	**0.76**	**0.27**	**0.21**	**2.81**	**0.005**

a. Dependent Variable: students’ quality of their participation in doing their group assignment

_**b**_. Dependent Variable: students’ attitudes towards their group assignment

^c^ 70 master students in medical school, who co-authored their group assignment on a wiki

All learners agreed or strongly agreed that the process of their co-constructive activities was motivating enough to apply their new knowledge and skills into their future academic lives.

## Discussion

In this action research, the impacts of the flipped classrooms on master students’ wiki-supported group assignment in a blended course were investigated. Exploring the synergistic effects of flipped classrooms and a wiki-authoring activity on students’ group-work, it was confirmed that a wiki-authoring GA is effective in achieving student learning outcomes. A wiki-authoring group assignment enhances students’ attitudes towards their GA and integrating a wiki with flipped classrooms increases wiki accomplishment.

In this study, the mean score of both the quantity and quality of students’ participation in doing their GA was significantly higher in the wiki-group, compared to those of the students’ in the non-wiki group (Sig = 0.000). Similar to our findings, in another study, students in wiki collaborative writing group had better performance in drafting and revising an argumentative essay, compared to the students in the face-to-face writing group [19].

Students in the wiki group were more satisfied with the whole course, compared to their peers in the non-wiki group (Sig = 0.000) too. Such a high satisfaction and quality in the wiki group can be related to the features of our wiki, tailored to the students’ needs and demands based on the findings from our own reflections and students’ feedback. This inference is in contrary to the findings of an action research at the Brunel University in England. In that research, none of the 75 participating undergraduate students had a wiki activity during the first 5 weeks of the study. After 5 weeks, 68% of students had just visited the wiki. According to the researchers, unsatisfying features of their wiki were the first and most important reason for their failure [11]. Moreover, the positive relationship between higher satisfaction of students and better quality of their GA in our wiki group could be credited not only to the satisfying and customized features of the wiki, but also to the integrative application of wiki with flipped classrooms.

Similarly, in a study conducted at Victoria University of Wellington, students in the online group allocated more time to the workgroup than the students in the face-to-face training group [8].

In a study at the University of Griffith in Australia, 180 first year students of psychology did their assignment for the course of the research and statistics on a wiki in groups of 4 to 6. The engagement rate of students in the wiki group was significantly higher than that of the control group; however, the overall student participation rate was not satisfactory from the viewpoint of the researchers because no reward had been considered for the participation of students in doing the homework on the wiki [20]. In our study, all students were informed that 40% of their overall score would be allocated to their GA. It seems that such a plan for assessment effectively derived students’ engagement and learning.

A wiki cannot be used to perform any type of GA and in any type of course. A course should require learners to work collaboratively. The activity in the wiki should be also trained and practiced. The courses at the postgraduate level are better choices to pilot wiki usage at universities [8]. Given these particulars, we used wiki for master students and for the course of writing articles in English, which requires collaborative learning. These points can be other reasons for the success in using wiki in our study.

In all, the effectiveness of our wiki-authoring activity in achieving student learning outcomes can be credited to satisfying and customized features of the wiki; a significant share of the end-of-semester score to the GA; integrative application of wiki with flipped classrooms; and employment of wiki for master students and for the course of writing articles in English, which requires collaborative learning.

The findings of this study lend evidence that integrating a wiki with flipped classrooms increases wiki accomplishment. In this study, unlike the students in the non-wiki group, students in the wiki group were significantly more satisfied with flipped classrooms (sig = 0.028). Furthermore, students’ satisfaction with flipped classrooms correlated at r = 0.485(Sig = 0.017) with quality of their participation in doing their GA for 70 students in the wiki group. The effectiveness of the FC approach in increasing students’ motivation and engagement is verified by a systematic review [21]; however, in another systematic review of the flipped classroom, a contradictory relationship between students’ satisfaction with FCs and their academic performance was reported. The second review was focused on nursing education and according to its authors, such a tension could be attributed to some features of the FC, like group-based activities in FCs [22].

In our study, students in the non-wiki group asked fewer questions and were less involved in the discussions on the forum. During the semester, they never asked for the teacher’s feedback. In order to complete their GA, they mostly paraphrased the text of the e-contents produced by the teacher, with poor evidence of critical thinking. These findings could justify the negative relationship between satisfactions with flipped classrooms with quality of the GA among students in the non-wiki group. In other words, students in the non-wiki group did not take advantages of collaborative learning and formative assessment for full. While, having more extrinsic motivation derived from peer learning and teacher feedback, students’ in the wiki group successfully monitored and regulated their learning processes and developed their meta-cognitive skills. These inferences are in line with the findings of a randomized experimental study, in which the model of positive and significant relations between motivation and formative assessment or meta-cognitive skills are confirmed [23]. According to findings of that study, “there is a significant positive effect of peer discussions combined with teacher feedback on both metacognition and motivation” [23]. Moreover, based on their self- declarations, each student in the non-wiki group did only one part of the GA. They did and submitted their GA at the end of the semester and they were not informed about the work done by their other teammates; therefore, they did not feel the need to get feedback from the course teacher during the semester; whereas, students in the wiki group collaboratively managed their GA with both team and personal accountabilities. All the team members as well as the course teacher had access to the content of the GA at all times and collaborative learning put them in a position of constructive competition to improve and enhance the quality of their work; therefore, most of the students in this group repeatedly requested feedback from the teacher”. These findings can be justified with the findings of Robertson and Fowler’s study in which medical students’ perceptions of learner-initiated feedback were elaborated. Based on those findings, students’ decision to request feedback is influenced by their performance. Students who start working and engage timely benefit from advantages of the teacher’s prompt and formative feedback; therefore, it is recommended to employ learner-centered feedback models where students are in the center of the model [24].

The findings of this study can be generalized to all millennial students, who are currently studying postgraduate. Given the generational differences between millennial students and Z generation of learners [25], it is recommended to investigate the effectiveness of wiki-authoring activities among students in the new generation Z students. Considering the point that generation Z students are changing our approaches to teaching and learning [26], they may be asked to upload voice files on the wiki (not to type any content).

### Limitations

Our study had some limitations. In this study, randomization was not considered at the time of the study design and before the data collection process;therefore, students had the right to decide which group they would like to work and they were free to choose one of the two available options for doing their group assignment. As volunteers might have a different interest in the subject of the study (either negative or positive); and might have been more motivated to learn about the study topic, the outcomes of this study might have been influenced by specific characteristics of the students in the wiki group (selection bias). To overcome the selection bias, it is recommended to collect the information about the students’ grade point average at the beginning of the course and to examine the results obtained in different categories, including good, average and weak students. Indeed, by asking more questions at the beginning of the study, student’s specific interests to learn more about the research topic as well as their particular characteristics can be explored and the findings can be adjusted for those potential confounders.

Moreover, in this study, students’ reaction to the educational intervention was assessed just by a validated satisfaction questionnaire. Applying exploratory research designs might address students’ perceptions, concerns and recommendations more and better in the future studies.

Our study was conducted in a single center (TUoMS) over eight semesters and it was focused on a single course. It is recommended to conduct similar studies in multiple centers/ institutions or on different academic courses as well.

## Conclusions

Based on the findings of this study, including an educational wiki in a blended course and holding the classes with an FC approach can increase wiki accomplishment. Such synergistic impacts of integrating a wiki-authoring activity with flipped classrooms improve both quality and quantity of group assignments among master students in the medical school. These findings approve the positive role of collaborative and constructivist learning in achieving greater learning outcomes for wiki-supported group assignments.

## Supplementary information

**Additional file 1 Appendix 1.** Satisfaction questionnaire.

**Additional file 2 Appendix 2**. Checklist to assess the quantity and quality* of student’s participation in doing group work.

## Data Availability

The wiki is not public but it is open only to the course students. The wiki and all the data, generated and analyzed in this study, will be available from the corresponding author upon any reasonable request.

## Abbreviations

CVI: Content Validity Index
CVR: Content Validity Ratio
FC: Flipped Classroom
ICC: Intra-class Correlation Coefficient
LMS: Learning Management System
Moodle: Modular Object-Oriented Dynamic Learning Environment
PHP: Hypertext Preprocessor
Q&As: Questions and Answers
SPSS: Statistical Package for the Social Sciences

## References

[1] Sanders D, Welk DS (2005). Strategies to scaffold student learning: applying Vygotsky's zone of proximal development. Nurse Educ.

[2] Slavin RE (2014). Cooperative learning and academic achievement: why does groupwork work. Anales de psicología.

[3] Roberts TS (2004). Online collaborative learning: Theory and practice.

[4] Roberts DH, Newman LR, Schwartzstein RM (2012). Twelve tips for facilitating Millennials’ learning. Med Teach.

[5] Schwartz L, Clark S, Cossarin M, Rudolph J. Educational wikis: Features and selection criteria. The International Review of Research in Open and Distributed Learning. 2004;5(1):1–6.

[6] Parker K, Chao J (2007). Wiki as a teaching tool. Interdisciplinary J E-learning Learning Objects.

[7] Trocky NM, Buckley KM (2016). Evaluating the impact of wikis on student learning outcomes: an integrative review. J Prof Nurs.

[8] Elgort I, Toland J, Smith AG (2008). Is wiki an effective platform for group course work?.

[9] Boulos MNK, Maramba I, Wheeler S (2006). Wikis, blogs and podcasts: a new generation of web-based tools for virtual collaborative clinical practice and education. BMC Med Educ.

[10] Lingard L, Albert M, Levinson W. Grounded theory, mixed methods, and action research. Bmj. 2008;337:459–61. 10.1136/bmj.39602.690162.47 18687728

[11] Cole M (2009). Using wiki technology to support student engagement: lessons from the trenches. Comput Educ.

[12] Masters K, Ellaway R (2008). E-learning in medical education guide 32 part 2: technology, management and design. Med Teach.

[13] Moodle. Available from: (https://docs.moodle.org/31/en/About_Moodle). (cited 2017 Dec(.

[14] Milman NB (2012). The flipped classroom strategy: what is it and how can it best be used?. Distance Learn.

[15] McLaughlin JE, Roth MT, Glatt DM, Gharkholonarehe N, Davidson CA, Griffin LM (2014). The flipped classroom: a course redesign to foster learning and engagement in a health professions school. Acad Med.

[16] Tatroe K, MacIntyre P, Lerdorf R (2013). Programming PHP: Creating Dynamic Web Pages: " O'Reilly Media, Inc.".

[17] Polit DF, Beck CT (2006). The content validity index: are you sure you know what's being reported? Critique and recommendations. Research in nursing & health.

[18] DeVon HA, Block ME, Moyle-Wright P, Ernst DM, Hayden SJ, Lazzara DJ (2007). A psychometric toolbox for testing validity and reliability. J Nurs Scholarsh.

[19] Ansarimoghaddam S, Hoon TB, Yong MF. Collaboratively Composing an Argumentative Essay: Wiki versus Face-to-face Interactions. GEMA Online® Journal of Language Studies. 2017;17(2):33–53.

[20] Neumann DL, Hood M. The effects of using a wiki on student engagement and learning of report writing skills in a university statistics course. Aust J Educ Technol. 2009;25(3):382–98.

[21] Chen F, Lui AM, Martinelli SM (2017). A systematic review of the effectiveness of flipped classrooms in medical education. Med Educ.

[22] Betihavas V, Bridgman H, Kornhaber R, Cross M (2016). The evidence for ‘flipping out’: a systematic review of the flipped classroom in nursing education. Nurse Educ Today.

[23] Molin F, Haelermans C, Cabus S, Groot W. The effect of feedback on metacognition-a randomized experiment using polling technology. Comput Educ. 2020;103885:1–21.

[24] Robertson AC, Fowler LC (2017). Medical student perceptions of learner-initiated feedback using a mobile web application. J Med Educ Curric Dev.

[25] Mohr KA, Mohr ES (2017). Understanding generation Z students to promote a contemporary learning environment. J Empower Teach Excellence.

[26] Hampton DC, Keys Y (2017). Generation Z students: will they change our nursing classrooms. J Nurs Educ Pract.

