# Supplementary material for: Assessment of the effect of application of an educational wiki in flipped classroom on students’ achievement and satisfaction
Source: BMC Med Educ. 2020 Sep 7;20:293. doi: 10.1186/s12909-020-02223-0 (PMC7487836; doi:10.1186/s12909-020-02223-0)
Supplement: Supplementary file 1 — Additional file 1 Appendix 1. Satisfaction questionnaire. [file 12909_2020_2223_MOESM1_ESM.pdf]

Appendix 1

Dear student,

Thanking you for completing this questionnaire, this study is aimed to explore the effects of your writing course, blended with flipped classrooms, on your attitude towards your wiki-supported group work and the quality of your group work. Please specify your satisfaction with each item by checking one of the options: very high, above the average, average, Average, below the average, very low. Completing the questionnaire will take about five minutes of your time. Your invaluable feedback will help us to improve the course. Be sure that a unique code will be assigned to you and all the research data will be saved confidential. You have the right to withdraw from the study whenever you want.

Kindest regards,
The research team

Component of educational intervention		Satisfaction item	The rate of the satisfaction				
			Very high	Above average	Average	Below average	Very low
**E-contents**	1	Fitness of the content of e-contents to my needs					
	2	The quality of e-contents					
**Forum**	3	The usefulness of forum discussions					
	4	User-friendliness of the forum discussions					
**Flipped classroom**	5	The effectiveness of the in- class discussions					
	6	Possibility to participate in class discussions					
	7	The worth of summary sharing in learning					
	8	The value of student presentations in learning					
	9	The role of teachers' brief lecture					
**Group work**	10	Appropriateness of the group work					
	11	The helpfulness of the group work					
**The whole course**		**Satisfaction with the whole course (self-declared)**					
**Attitude toward performing their group**	1	The value of group work in motivating me to learn more					
	2	The impact of the group work to increase my confidence in writing an article					
	3	The worth of the group work in identifying my weaknesses and strengths in writing articles					
	4	The importance of the group work experience in building my teamwork skills					
	5	The role of the group work in encouraging me to participate in similar experiences					

Please rate your satisfaction with the quiz at the beginning of the classes by specifying a number from 1 to 5: Your rating: ☐

Please rate your satisfaction with the quiz at the beginning of the classes by specifying a number from 1 to 5: Your rating: ☐
