# Supplementary material for: Assessment of the effect of application of an educational wiki in flipped classroom on students’ achievement and satisfaction
Source: BMC Med Educ. 2020 Sep 7;20:293. doi: 10.1186/s12909-020-02223-0 (PMC7487836; doi:10.1186/s12909-020-02223-0)
Supplement: Supplementary file 2 — Additional file 2 Appendix 2. Checklist to assess the quantity and quality* of student’s participation in doing group work. [file 12909_2020_2223_MOESM2_ESM.pdf]

Appendix 2

Checklist to assess the quantity and quality* of student's participation in doing group work

Student name& number:

The way of doing group work (GW): On Wiki☐ In a traditional way (paper based)☐

Assessment dimension	Assessment item	Rating					
		0	1	2	3	4	5
Quantity of student's participation in doing GW	Quantity of participation in writing text for GW						
	Quantity of participation in editing their own text						
	Quantity of participation in editing their peers' text						
	Quantity of participation in structuring and organization of the text						
	Quantity of participation in importing files and hyperlinking of valid references						
Quality of student's participation in doing GW	Quality of participation in writing text for GW						
	Quality of participation in editing their own text						
	Quality of participation in editing their peers' text						
	Quality of participation in structuring and organization of the text						
	Quality of participation in importing files and hyperlinking of valid references						

Score of the quantity of the participation in doing GA= $\frac{Sum\ of\ ratings\ of\ all\ five\ items\ of\ quantity}{5}$

Score of the quality of the participation in doing GA= $\frac{Sum\ of\ ratings\ of\ all\ five\ items\ of\ quality}{5}$

Student's quantity score= ☐

Student's quality score= ☐

* **The criteria to assess the quality:** completeness, accuracy and pertinence of the content, which is written by each student. The usage of pertinent terminologies and the quality of the links established between the pages developed by other students and their own pages
